# National Cancer Database Comparison of Radical Cystectomy vs Chemoradiotherapy for Muscle‐Invasive Bladder Cancer: Implications of Using Clinical vs Pathologic Staging

**DOI:** 10.1002/cam4.1684

**Published:** 2018-10-10

**Authors:** Hong‐Yiou Lin, Hong Ye, Kenneth M. Kernen, Jason M. Hafron, Daniel J. Krauss

**Affiliations:** ^1^ Department of Radiation Oncology Beaumont Health Royal Oak Michigan; ^2^ Michigan Institute of Urology and Beaumont Health Troy Michigan

**Keywords:** bladder cancer, chemotherapy, National Cancer Database, radiation, radical cystectomy

## Abstract

**Purpose:**

To test the hypothesis that bladder preservation therapy consisting of definitive chemoradiotherapy (chemoRT) results in similar overall survival rates to radical cystectomy/chemotherapy when balancing baseline patient characteristics and initial (preoperative) clinical stage.

**Materials/methods:**

A total of 7,322 patients with stage II‐IV, M0 bladder cancer who were treated with cystectomy/chemo (N = 5,664) or definitive chemoRT (N = 1,658) were identified from the National Cancer Database. Baseline patient characteristics were compared using Pearson's chi‐square, Fisher's exact test, and Wilcoxon's rank sum tests. Cox regressions were used to investigate for variables significantly correlated with overall survival (OS). OS was compared between cystectomy/chemo vs chemoRT before and after propensity score matched pair analyses using Kaplan‐Meier curves and log‐rank tests.

**Results:**

Patients who underwent cystectomy/chemo were significantly younger than ones treated with definitive chemoRT (mean age 63.7 vs 75.2; *P *<* *0.001). Age, race, Charlson/Deyo Comorbidity Score (CDCS), clinical stage, insurance status, and type of facility significantly correlated with OS (*P* < 0.05 for all covariates). Patients treated with cystectomy/chemo were younger, healthier with better CDCS, and more likely treated at academic facilities. Before matched pair analyses, OS was significantly better when treated with cystectomy/chemo (3 year 56.4%; 5 year 45.9%) compared to chemoRT (3 year 47.3%; 5 year 33.2%) (*P* < 0.001); 28.6% of patients undergoing cystectomy were upstaged at the time of surgery. After matched pair analyses matching age, race, sex, CDCS, clinical (presurgical) stage, insurance, and facility type (N = 1,750), OS was no longer significantly different between cystectomy/chemo (3 year 52.1% and 5 year 41.0%) vs chemoRT (3 year 53.3% and 5 year 40.1%) (*P* = 0.5).

**Conclusions:**

Patients treated with cystectomy/chemo were significantly younger and healthier compared to those treated with chemoRT. Once these factors were accounted for in propensity score matched pair analyses using clinical stage, overall survival was not significantly different between cystectomy/chemo and an organ‐sparing approach with definitive chemoRT.

## 1 INTRODUCTION

Bladder cancer is the 6th most common cancer in the United States with an estimated 79,030 new cases responsible for 16,870 deaths in 2017.^1^ Internationally, bladder cancer is also a major cause of morbidity and mortality as the 9th most common malignancy.^2^ The majority of bladder cancers are non‐muscle‐invasive, but approximately 21% of bladder cancers are muscle‐invasive at diagnosis.^3^, ^4^, ^5^ According to the NCCN, category 1 recommendation for the treatment of muscle‐invasive bladder cancer is neoadjuvant cisplatin‐based combination chemotherapy followed by radical cystectomy with bilateral pelvic lymph node dissection and urinary diversion.^6^ Unfortunately, radical cystectomy is associated with significant perioperative morbidity and mortality with 67% of patients experiencing complications and up to 2% death rate within 90 days of surgery.^7^

Bladder preservation therapy consisting of transurethral resection of bladder tumor (TURBT) followed by combination chemoradiotherapy (chemoRT) is an alternative treatment to cystectomy for muscle‐invasive bladder cancer.^6^, ^8^ While there has not been a prospective randomized trial comparing neoadjuvant chemotherapy followed by radical cystectomy with the organ‐sparing approach, multiple prospective randomized and nonrandomized definitive chemoRT trials have shown overall survival rates that are comparable to radical cystectomy trials.^9^, ^10^, ^11^, ^12^, ^13^, ^14^, ^15^ In the absence of prospective randomized trials comparing cystectomy with definitive radiotherapy, investigators have used both institutional and large national databases including Surveillance, Epidemiology, and End Results (SEER) and the National Cancer Database (NCDB) to compare treatment outcomes.

While multiple SEER and NCDB analyses have reported better overall survival when patients were treated with radical cystectomy compared to definitive chemoRT, those studies have been confounded by imbalances in patient characteristics.^16^, ^17^, ^18^, ^19^, ^20^ Attempts have been made to adjust for these differences using matched pair analyses, but analytic stage (matching the pathologic stage of cystectomy with the clinical stage of chemoRT patients) has been chosen for such comparisons in the past.^17^ The “upstaging” of surgical patients is yet another potential confounder when this is done. Other previous matched pair analyses were limited by the low radiation dose used to specify definitive chemoRT patients (median dose 45 Gy) or limiting the comparison to octogenarians.^20^, ^21^ As institutional retrospective matched pair analyses of cystectomy and chemoRT have shown similar survival outcomes when clinical stage was used for matching, we aimed to confirm this in the context of a large, population‐based cohort using the NCDB.^22^ We hypothesized that balancing baseline patient characteristics with propensity score matched pair analyses using clinical stage would show similar overall survival between radical cystectomy with chemotherapy and definitive chemoRT.

## 2 METHODS AND MATERIALS

### 2.1 Data source

We extracted data from the National Cancer Database. The NCDB is a national cancer registry maintained by the American College of Surgeons’ Commission on Cancer and the American Cancer Society. Patient data are sourced from hospital registry data and collected from more than 1500 Commission on Cancer accredited facilities. More than 70% of newly diagnosed cancer cases nationwide are included in the NCDB.^23^ This research was performed in compliance with our Institutional Review Board guidelines.

### 2.2 Cohort selection

**Figure 1:**
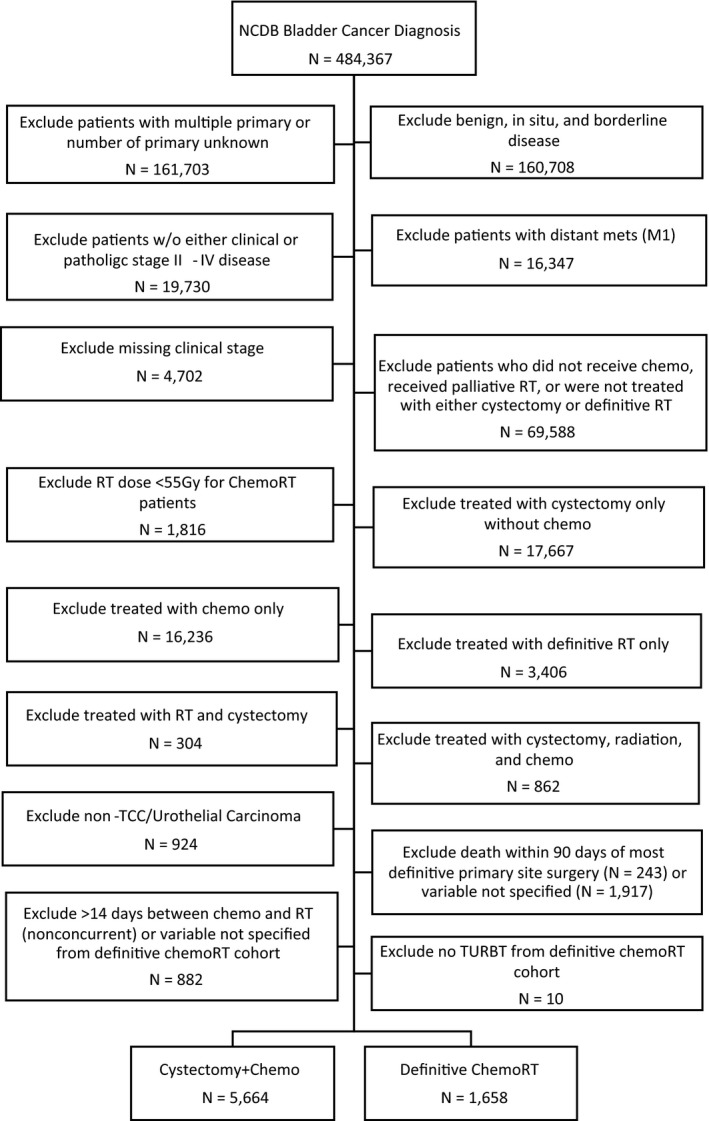
Consolidated Standards of Reporting (CONSORT) diagram illustrating the selection criteria for the cystectomy/chemo and definitive chemoRT groups. ChemoRT, chemoradiotherapy. Chemo, chemotherapy. RT, radiotherapy

484,367 patients were identified from the NCDB who had *International Classification of Disease for Oncology, 3rd Edition* (ICD‐O‐3) site codes C67.0 to C67.9 corresponding to a diagnosis of urinary bladder malignancy (Figure 1). All patients received treatment between 2004 and 2014. Histology was limited to only transitional cell carcinoma/urothelial carcinoma or its variants (histology code 8120, 8122, 8130, and 8131). Radical cystectomy was defined as radical cystectomy; radical cystectomy plus either ileal conduit, continent reservoir/pouch, abdominal pouch, or in situ pouch; pelvic exenteration NOS; or radical cystectomy including anterior exenteration, posterior exenteration, or total exenteration (surgery to primary site code 60‐74). The total radiation dose was the summation of the regional dose (RAD_REGIONAL_DOSE_CGy) and boost dose (RAD_BOOST_DOS_CGy). The regional dose and boost dose variables were reviewed for each patient individually, and patients with unknown radiation doses were excluded. All definitive chemoRT patients were required to have a radiation dose ≥55 Gy, chemotherapy administered within 14 days of radiotherapy start date (DX_CHEMO_STARTED_DAYS and DX_RAD_STARTED_DAYS within 14 days of each other), and had TURBT (RX_SUMM_SURG_PRIM_SITE codes 10, 12, 14, 20, 25, 26, and 27). Palliative RT was defined as dose ≤30 Gy. To account for immortal time bias, patients who died within 90 days of the most definitive primary site surgery were excluded (PUF_90_DAY_MORT_CD). Chemotherapy was defined as chemotherapy administered with the first course of therapy (RX_SUMM_CHEMO code 01‐03) for both radical cystectomy and chemoRT patients. For the cystectomy/chemo cohort, neoadjuvant, adjuvant, or both neoadjuvant and adjuvant chemotherapy were defined using the “RX_SUMM_SYSTEMIC_SUR_SEQ” variable using codes 2 to 4. Immunotherapy was not considered to be chemotherapy.

Patients who did not receive chemo, had no treatment details about cystectomy or RT, not treated with either cystectomy or definitive RT, or received palliative RT (≤30 Gy) were excluded from the study cohort (N = 69,588). Additionally, patients who had more than one primary and unknown number of primary (N = 161,703), non‐invasive disease (N = 160,708), without either pathologic/clinical stage II‐IV disease (N = 19,730), with distant metastases (N = 16,347), missing clinical stage (N = 4,702), RT dose <55 Gy for chemoRT patients (N = 1,816), treated with cystectomy without chemotherapy (N = 17,667), treated with chemotherapy only (N = 16,236), treated with definitive RT only (N = 3,406), treated with both definitive RT and cystectomy (N = 304), treated with trimodality (cystectomy, radiation, and chemo) therapy (N = 862), without TURBT in the definitive chemoRT cohort (N = 10), non‐TCC/urothelial carcinoma histology (N = 924), chemotherapy not administered concurrently with radiotherapy in the definitive chemoRT cohort (N = 882), and patients who died or had unknown survival within 90 days of the most definitive primary site surgery (N = 2,160) were excluded from analysis. With the above exclusions, there were 5,664 chemo/cystectomy and 1,658 definitive chemoRT patients that were studied in the cohort (Figure 1).

### 2.3 Covariates

Patient characteristics including age, sex, race, Charlson/Deyo Comorbidity Score (CDCS), clinical stage, analytic stage, insurance, income, tumor grade, radiation dose, overall survival, and type of treatment facility were extracted from the NCDB to analyze for variables that significantly correlated with overall survival. Race was recoded to white, black, and other given the small number of patients that were neither black/white and the large number of racial categories. Clinical and pathologic stages were recoded as stage 0, I, II, III, and IV by combining the sub‐stages within each stage. CDCS was recoded as 0, 1, and 2 or more. Overall survival was analyzed using the “dx_lastcontact_death_months” variable, which indicated the number of months between the date of diagnosis and the date of death or last contact. For comparison of insurance status, public insurance included Medicare, Medicaid, and other government.

### 2.4 Statistical analysis and propensity score matched pair analyses

Associations between treatment modality and patient or facility characteristics were evaluated using Pearson's chi‐square or Fisher's exact test for categorical and Wilcoxon's rank sum test for ordinal and continuous data. Univariate and multivariate Cox regressions were used to assess for variables significantly correlated with overall survival (OS) in the unmatched and propensity score matched pair cohort. Patient variables significantly associated with overall survival on multivariate analyses with a threshold of *P* < 0.05 or deemed clinically important (ie, sex) were included into propensity score matched pair models. Patients treated with cystectomy/chemo were matched 1:1 to patients treated by definitive chemoRT. A propensity score tolerance of 0 was chosen for matched pair analyses to compare cystectomy/chemo and definitive chemoRT. Both analytic stage and clinical stages were used for propensity score matching. Overall survival was compared between cystectomy/chemo vs chemoRT both before and after propensity score matched pair analyses using Kaplan‐Meier curves and log‐rank tests. All statistical analyses were conducted using SPSS 24 (IBM Corp. Released 2016. IBM SPSS Statistics for Windows, Version 24.0. Armonk, NK: IBM Corp).

## 3 RESULTS

### 3.1 Cohort selection and patient characteristics

5,664 patients treated with cystectomy/chemo and 1,658 treated with combination chemoRT met study criteria and were included in the analysis. Baseline patient characteristics were imbalanced between cystectomy/chemo and chemoRT cohorts (Table 1). Patients in the cystectomy/chemo group were significantly younger (difference in mean age of 11.5 years; *P* < 0.001), healthier with lower CDCS (*P* < 0.001), treated at academic facilities (*P* < 0.001), and had more private insurance (*P* < 0.001). There were no significant differences in race and income between cystectomy/chemo and chemoRT.

**Table 1:** Patient characteristics before propensity score matched pair analysis

	Cystectomy and Chemo	RT and Chemo	*P* value
Year of treatment	2004‐2014	2004‐2014	
Number of patients	5664	1658	
Mean follow‐up time (mo)	36.6	33.4	**<0.001**
Age			
Mean	63.7	75.2	**<0.001**
Median	64	77	
Sex			
Male	3818 (67.4%)	1251 (75.5%)	**<0.001**
Female	1846 (32.6%)	407 (24.5%)	
Race			
White	5137 (90.7%)	1490 (89.9%)	0.800
Black	316 (5.6%)	117 (7.0%)	
Other/unknown	211 (3.7%)	51 (3.1%)	
Charlson/Deyo Comorbidity Score (CDCS)			
0	4227 (74.6%)	1097 (66.1%)	**<0.001**
1	1172 (20.7%)	396 (23.9%)	
2 or more	265 (4.7%)	165 (10.0%)	
Clinical stage			
II	3697 (65.3%)	1272 (76.7%)	**<0.001**
III	1085 (19.1%)	252 (15.2%)	
IV	882 (15.6%)	134 (8.1%)	
Analytic stage^a^			
II	2077 (36.7%)	1269 (76.5%)	**<0.001**
III	1509 (26.6%)	252 (15.2%)	
IV	2078 (36.7%)	137 (8.3%)	
Insurance			
Private	2426 (42.8%)	297 (17.9%)	**<0.001**
Medicare	2522 (44.5%)	1235 (74.5%)	
Uninsured	211 (3.7%)	35 (2.1%)	
Medicaid	322 (5.7%)	53 (3.2%)	
Other government	65 (1.1%)	26 (1.6%)	
Unknown	118 (2.1%)	12 (0.7%)	
Income			
<$30, 000	551 (9.7%)	189 (11.4%)	0.174
$30, 000‐$34, 999	1043 (18.4%)	314 (18.9%)	
$35, 000‐$45, 999	1584 (28.0%)	467 (28.2%)	
≥$46, 000	2260 (39.9%)	629 (37.9%)	
Unknown	226 (4.0%)	59 (3.6%)	
Grade			
Well differentiated	35 (0.6%)	11 (0.7%)	**<0.001**
Moderately differentiated	121 (2.1%)	71 (4.3%)	
Poorly differentiated	2491 (44.0%)	808 (48.7%)	
Undifferentiated	2341 (41.3%)	590 (35.6%)	
Unknown	676 (11.9%)	178 (10.7%)	
Facility type			
Community cancer program	413 (7.3%)	229 (13.8%)	**<0.001**
Comprehensive CCP	1783 (31.5%)	823 (49.6%)	
Academic	2825 (49.9%)	409 (24.7%)	
Integrated network cancer program	586 (10.3%)	196 (11.8%)	
Other/unknown	57 (1%)	1 (0.06%)	
Radiation dose			
Mean	N/A	6409 cGy	N/A
Median	N/A	6480 cGy	
Mode	N/A	6480 cGy	

a Pathologic stage assigned but substituted with clinical stage if pathologic stage unavailable.

Bolded values are statistically significant at *P* < 0.05.

Analytic stage is a variable specified by the NCDB. Analytic stage preferentially assigns pathologic stage but substitutes with clinical stage if pathologic stage is unavailable. Given that pathologic stage is unavailable for chemoRT patients, the clinical stage and analytic stage are almost identical for the chemoRT cohort (Table 1). In contrast, there are significant differences between the overall TNM clinical and analytic stages for the cystectomy/chemo group as upstaging occurs in 1,620 (28.6%) patients. Specifically, 424 (7.5%) patients were upstaged to pathologic overall stage III and 1,196 (21.1%) patients were upstaged to pathologic overall stage IV after cystectomy. In the cystectomy/chemo cohort, the number of node‐positive patients increased by 1,145 (20.2%) after radical cystectomy.

### 3.2 Variables significantly correlated with overall survival

To investigate for variables significantly correlated with overall survival prior to propensity score matched pair analyses, univariate and multivariate Cox regressions were performed (Table 2). Variables significantly correlated with overall survival were age (*P* < 0.001), race (*P* < 0.004), CDCS (*P* < 0.001), clinical stage (*P* < 0.001), facility type (*P* < 0.008), and insurance status (*P* = 0.019; Table 2). Sequencing of chemotherapy with surgery (*P* < 0.001) and radiation dose (*P* < 0.001) significantly correlated with overall survival for the cystectomy/chemo and definitive chemoRT patients, respectively (Table 2).

**Table 2:** Patient variables significantly correlated with survival prior to propensity score matched pair analyses

	Univariate		Multivariate	
	*P* value	Hazard ratio (95% CI)	*P* value	Hazard ratio (95% CI)
Age	**<0.001**	1.019 (1.016‐1.022)	**<0.001**	1.012 (1.008‐1.017)
Sex (reference: male)				
Female vs Male	0.073	1.065 (0.994‐1.141)	0.322	1.040 (0.962‐1.124)
Race (reference: white)				
Black vs White	**0.001**	1.244 (1.093‐1.415)	**<0.001**	1.295 (1.125‐1.491)
Other vs White	**0.006**	0.703 (0.547‐0.904)	**0.004**	0.676 (0.519‐0.882)
Unknown vs White	**0.037**	0.729 (0.541‐0.982)	0.213	0.811 (0.583‐1.128)
Charlson/Deyo Comorbidity Score (reference: 0)				
1 vs 0	**<0.001**	1.230 (1.139‐1.328)	**<0.001**	1.174 (1.080‐1.276)
≥2 vs 0	**<0.001**	1.521 (1.341‐1.724)	**<0.001**	1.396 (1.220‐1.598)
Clinical stage (reference: II)				
III vs II	**<0.001**	1.273 (1.173‐1.383)	**<0.001**	1.309 (1.197‐1.433)
IV vs II	**<0.001**	1.647 (1.511‐1.795)	**<0.001**	1.827 (1.661‐2.010)
Facility type (reference: Non‐academic)				
Academic vs Non‐academic	**<0.001**	0.822 (0.770‐0.877)	**0.008**	0.906 (0.842‐0.974)
Treatment modality (reference: cystectomy/chemo)				
RT chemo vs Cystectomy/chemo	**<0.001**	1.473 (1.372‐1.582)	**0.006**	1.144 (1.039‐1.259)
Insurance (reference: 2nd variable)				
Private vs Not insured/unknown	0.052	0.857 (0.733‐1.002)	0.104	0.869 (0.734‐1.029)
Government vs Not insured/unknown	**0.029**	1.184 (1.018‐1.378)	0.677	0.963 (0.807‐1.149)
Government vs Private	**<0.001**	1.383 (1.290‐1.481)	**0.019**	1.112 (1.018‐1.214)
Income (reference: <$30,000)				
$30,000‐$34,999 vs <$30,000	0.355	1.060 (0.937‐1.198)	0.330	1.069 (0.935‐1.223)
$35,000‐$45,999 vs <$30,000	0.285	0.939 (0.836‐1.054)	0.601	0.966 (0.850‐1.098)
≥$46,000 vs <$30,000	**0.008**	0.860 (0.769‐0.962)	0.085	0.896 (0.790‐1.015)
Grade (reference: Well differentiated)				
Moderately vs Well differentiated	0.339	1.269 (0.778‐2.070)	0.195	1.443 (0.829‐2.514)
Poorly vs Well differentiated	0.112	1.443 (0.918‐2.268)	**0.035**	1.731 (1.040‐2.882)
Undifferentiated vs Well differentiated	0.239	1.312 (0.835‐2.064)	**0.049**	1.668 (1.002‐2.779)
Unknown vs Well differentiated	0.339	1.252 (0.790‐1.986)	0.056	1.659 (0.987‐2.787)
Chemo sequencing (for cystectomy/chemo patients only) (Reference: neoadjuvant)				
Adjuvant vs Neoadjuvant	**<0.001**	1.425 (1.303‐1.558)	**<0.001**	1.353 (1.236‐1.482)
Both adjuvant/neoadjuvant vs Neoadjuvant	**0.047**	1.164 (1.002‐1.352)	0.073	1.150 (0.987‐1.340)
Radiation dose (for chemoRT patients only) (Reference:≥60 Gy)				
55‐59.9 Gy vs ≥60 Gy	**<0.001**	1.588 (1.338‐1.884)	**<0.001**	1.681 (1.379‐2.049)

MVA, multivariate analysis.

Bolded values are statistically significant at *P* < 0.05.

### 3.3 Survival before propensity score matched pair analyses

**Figure 2:**
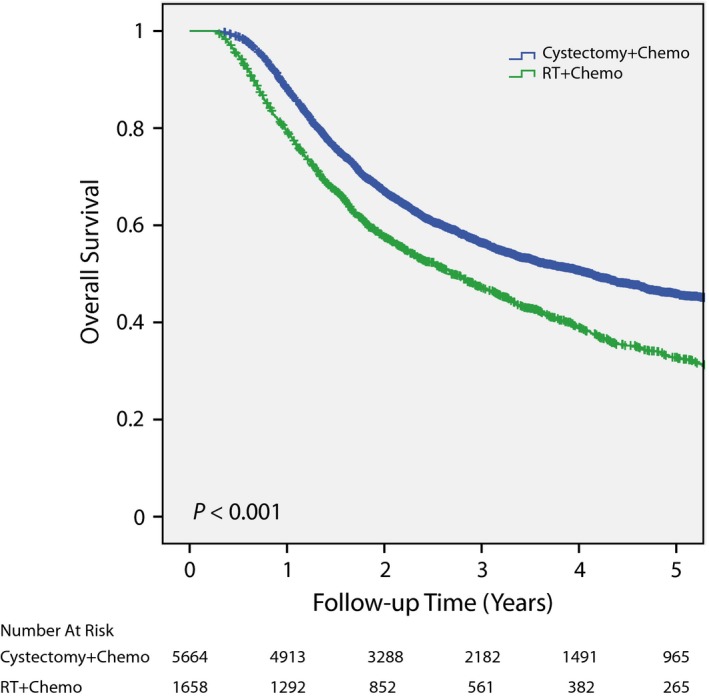
Overall survival before matched pair analysis. Prior to a matched pair analysis, patients treated with cystectomy/chemo had significantly better overall survival compared to definitive chemoRT. Chemo, chemotherapy. RT, radiotherapy.

Before matched pair analyses, patients treated with cystectomy/chemo had significantly better overall survival compared to chemoRT (*P* < 0.001; Figure 2). The actuarial 3 year OS was 56.4% (95% CI 55.0%‐57.8%) and 47.3% (95% CI 44.8%‐49.8%) for the cystectomy/chemo and chemoRT cohorts, respectively. The actuarial 5 year OS was 45.9% (95% CI 44.3%‐47.5%) and 33.2% (95% CI 30.5%‐35.9%) for the cystectomy/chemo and chemoRT groups, respectively.

### 3.4 Survival after propensity score matched pair analyses using clinical stage vs analytic stage

**Figure 3:**
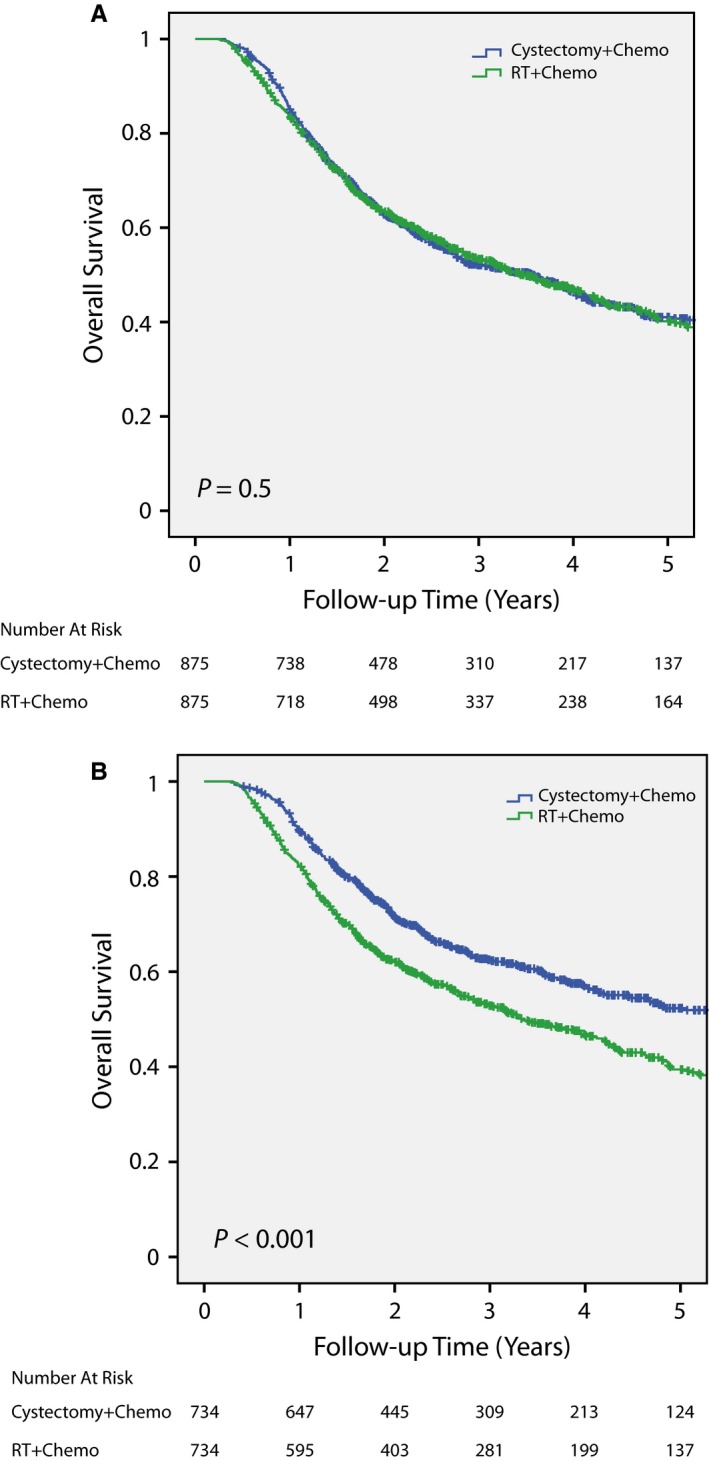
Overall survival after matched pair analysis using either clinical stage (A) or analytic stage (B). There were no significant differences in survival between cystectomy/chemo and chemoRT patients after a matched pair analysis using clinical stage (Figure 3A). However, matched pair analysis using analytic stage showed better overall survival for cystectomy/chemo patients (Figure 3B).

To investigate whether differences in patient survival between cystectomy/chemo and chemoRT could be attributed to imbalance in baseline patient characteristics, propensity score matched pair analyses were conducted. The most stringent matched pair analyses matching age, race, sex, CDCS, clinical stage, insurance, and facility type resulted in 875 pairs with 1,750 total patients (Figure 3A; Table S1A for patient characteristics after matched pair analysis). Matching for these factors, OS became similar between cystectomy/chemo and chemoRT patients (*P* = 0.500). The actuarial 3‐ and 5‐year overall survival rates were 52.1% (95% CI 48.6%‐55.6%) and 41.0% (95% CI 37.1%‐44.9%) for cystectomy/chemo patients compared to 53.3% (95% CI 49.8%‐56.8%) and 40.1% (95% CI 36.4%‐43.8%) for chemoRT patients, respectively.

We also performed a matched pair analysis similar to the one above matching age, race, sex, CDCS, analytic stage (instead of clinical stage), insurance, and facility type resulting in 734 pairs with 1,468 patients. In contrast to matching based on clinical stage, matching with analytic stage showed better overall survival for patients treated with cystectomy/chemo than chemoRT (*P* < 0.001, Figure 3B;Table S1B for patient characteristics). In summary, keeping all other matching criteria identical, a matched pair analysis using clinical stage showed similar overall survival between cystectomy/chemo and chemoRT while a second matched pair analysis based on analytic stage showed better overall survival for the cystectomy/chemo cohort.

After propensity score matched pair analyses, Cox univariate and multivariate regressions were conducted to assess for variables significantly correlated with overall survival. Treatment modality was not significantly correlated with overall survival after propensity score matched pair analyses using clinical stage, but modality was significantly correlated with overall survival after matched pair analyses utilizing analytic stage as well as in the unmatched cohort (Tables 2 and S2).

### 3.5 Propensity score matched pair analyses with progressively less stringent matching criteria

To investigate whether the similarity in survival outcomes between cystectomy/chemo and chemoRT after propensity score matched pair analyses were attributable to stringent selection criteria resulting in small sample sizes, we performed additional matched pair analyses with progressively less stringent matching criteria.

Propensity score matched pair analysis matching age, sex, CDCS, clinical stage, insurance, and facility type resulted in 978 pairs with 1,956 patients. There were no differences in overall survival between cystectomy/chemo and chemoRT (Table S1A, *P* = 0.303;Table S3A for patient characteristics after matched pair analysis).

A less stringent yet matched pair analysis matching age, CDCS, clinical stage, insurance, and facility type resulted in 1,059 pairs with 2,118 patients. There were also no differences in overall survival between cystectomy/chemo and chemoRT (Figure S1B, *P* = 0.124; Table S3B for patient characteristics).

To further increase sample size, chemoRT patients who did not receive concurrent chemoRT or TURBT were included in the chemoRT cohort and matched with cystectomy/chemo matching age, race, sex, CDCS, clinical stage, insurance, and facility type. There were 1,270 pairs with 2,540 patients, and no significant differences were seen in overall survival between cystectomy/chemo and chemoRT (Figure S1C, *P* = 0.357; Table S3C for patient characteristics).

### 3.6 Effect of chemotherapy sequencing and radiation dose on overall survival

**Figure 4:**
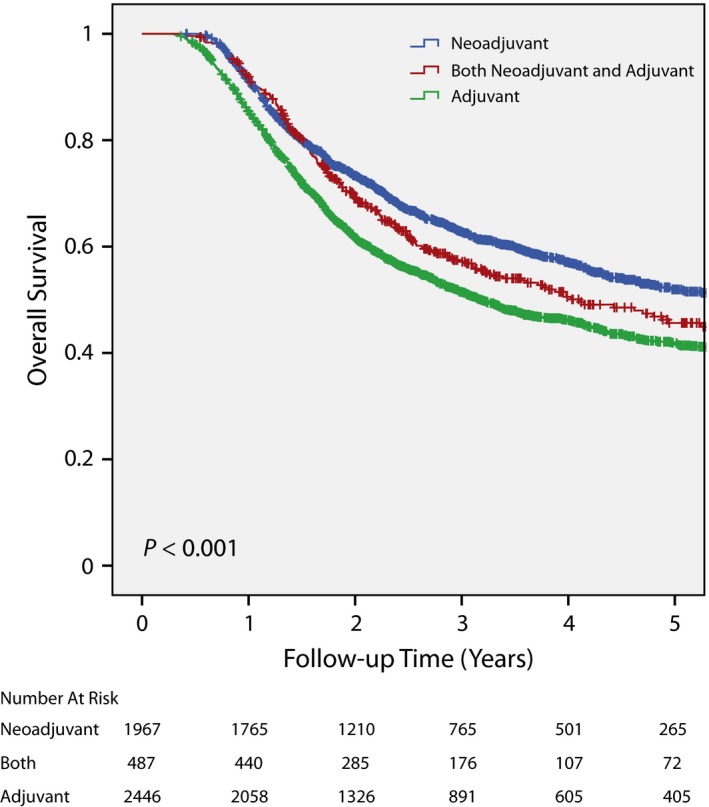
Overall survival in the cystectomy/chemo cohort stratified by when chemotherapy was administered relative to cystectomy. Patients treated with neoadjuvant chemotherapy had significantly better overall survival compared to ones treated with adjuvant chemotherapy or both neoadjuvant and adjuvant chemotherapy.

For cystectomy/chemo patients, the effect of sequencing between systemic therapy and surgery on overall survival was investigated. Neoadjuvant systemic therapy was found to have better overall survival compared to adjuvant or both neoadjuvant and adjuvant systemic therapy (*P* < 0.001; Figure 4).

**Figure 5:**
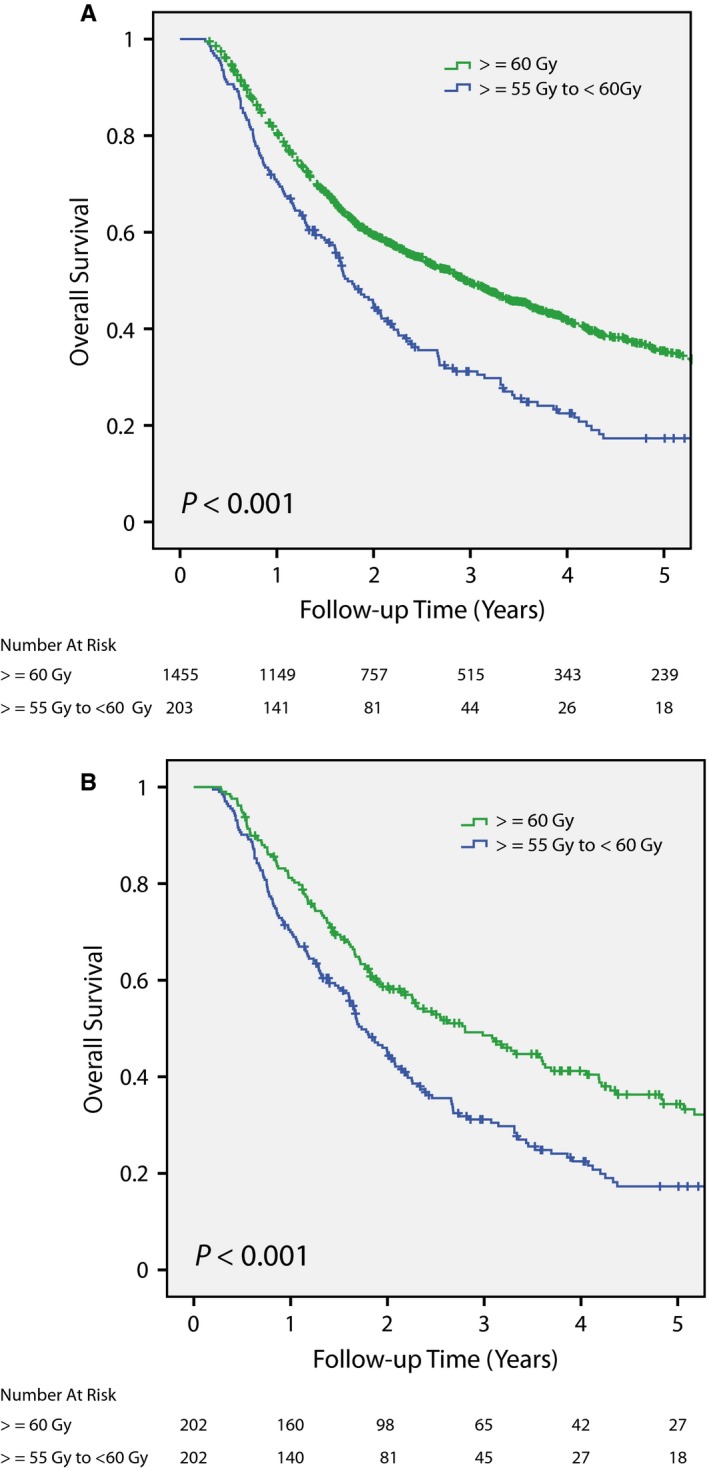
Overall survival in the definitive chemoRT cohort stratified by radiation dose before (A) and after (B) matched pair analyses. Definitive chemoRT patients who received radiation dose ≥60 Gy had better overall survival than ones treated with ≥55 to <60 Gy of radiotherapy before matching baseline patient characteristics. After matching baseline patient characteristics, patients treated with radiation dose ≥60 Gy continued to show better overall survival.

For the chemoRT cohort, whether radiation dose had a significant effect on overall survival was analyzed. Patients treated with definitive chemoRT radiation doses ≥60 Gy had significantly better overall survival compared to ones who received ≥55 Gy to <60 Gy both before and after matched pair analyses (*P* < 0.001; Figure 5A,B;Table S4A,B).

**Figure 6:**
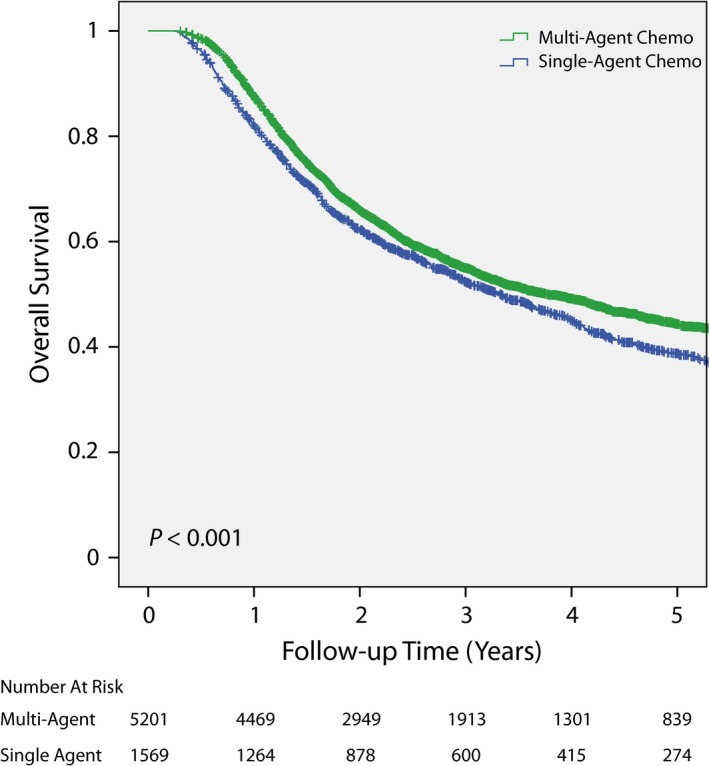
Comparison of overall survival based on single vs multiagent chemotherapy. Patients treated with multiagent chemotherapy had better overall survival than ones who received single‐agent chemotherapy. Chemo, chemotherapy.

The effect of single vs multiagent chemotherapy administered with cystectomy/chemo or definitive chemoRT on overall survival was also investigated. Patient who received multiagent chemotherapy had better overall survival compared to ones treated with single‐agent chemotherapy (*P* < 0.001; Figure 6).

## 4 DISCUSSION

Patients treated with radical cystectomy and chemotherapy were younger and had better CDCS compared to their chemoRT counterparts. Overall survival was better in patients treated with cystectomy/chemo than chemoRT prior to propensity score matched pair analyses or when patients were matched according to analytic stage. Upstaging occurred in 28.6% of cystectomy/chemo patients. However, matching using overall clinical stage instead of analytic stage showed similar overall survival between cystectomy/chemo and chemoRT.

This report provides a possible explanation as to why prior large database analyses have shown better overall survival for bladder cancer patients treated with cystectomy/chemo compared to definitive chemoRT whereas prospective multi‐institution radiotherapy trials have shown overall survival comparable to radical cystectomy—use of analytic stage instead of clinical stage in prior NCDB matched pair analyses.^17^, ^18^, ^19^, ^20^, ^22^, ^24^ Given that only clinical stage is available to the patient and oncologists prior to definitive local therapy, clinical stage represents a more accurate comparison between the two treatment approaches. One advantage of NCDB compared to SEER is that clinical and pathologic stages are coded separately, which allowed for separate matching based on clinical or pathologic stage and the ability to account for upstaging. SEER did not code for analytic stage prior to 2015 (separate AJCC clinical and pathologic stage added in 2015).^25^

Bekelman et al.^26^ used SEER to compare survival after treatment with either cystectomy or definitive radiotherapy and found that cystectomy patients had better overall survival as well as cancer‐specific survival in an unmatched comparison. Bekelman et al. then used estimated rates of upstaging from the literature to compare survival between cystectomy and radiotherapy. After the above multivariate adjustments, the authors found no difference in survival between cystectomy and definitive radiotherapy in a Cox proportional hazards regression. Thus, when estimated upstaging rates were accounted for in SEER, there were no differences in survival between cystectomy and radiotherapy. Using the NCDB, this manuscript showed cystectomy/chemo upstaging rates to be 28.6%. In this report, clinical stage was used to match between cystectomy and radiotherapy instead of upstaging estimates from the literature.

When matched based on analytic stage, the finding that patients treated with radical cystectomy had better overall survival than chemoRT was in agreement with a published report using the NCDB.^17^ Cahn et al. showed that cystectomy resulted in improved overall survival compared with definitive radiotherapy in an unmatched comparison as well as in matched pair analyses using analytic stage. Matching based on analytic stage entails matching the pathologic stage of cystectomy patients with the clinical stage of chemoRT ones. This did not take into account possible upstaging for chemoRT patients. In fact, upstaging from a lower clinical stage to a higher pathologic stage occurred in 28.6% of the cystectomy/chemo patients. Comparing the pathologic stage of cystectomy patients with the clinical stage of chemoRT patients would not take into account the possibility that close to 1/3 of the chemoRT patients most likely had a higher stage. Thus, a matched pair analysis based on analytic stage would likely understage the extent of disease for many patients treated with chemoRT, thereby introducing a selection bias that would result in the illusion of worse overall survival for noncystectomy patients.

This investigation is limited by its retrospective nature, statistical limitations inherent from propensity score matched pair analyses, inability to verify individual patient's treatment, lack of randomization, patient selection bias/confounding factors, unable to account for all possible variables, and exclusion of patients with missing covariates central to a comparison between cystectomy/chemo vs definitive chemoRT. Due to differences in baseline patient characteristics, using tolerance scores of 0.2, 0.1, 0.05, 0.03, 0.01, and 0.001 all continued to show significant differences in clinical stage between cystectomy and chemoRT patients. As a result, a tolerance score of 0 was chosen for our propensity score matched pair analyses, which meant that approximately 2/3 of the patients could be matched between cystectomy and chemoRT. To compensate for this decrease in matched patients, we performed additional matched pair analyses using progressively less stringent matching criteria as well as including the chemoRT patients that did not receive TURBT or concurrent chemotherapy—there were again no differences in overall survival between cystectomy and chemoRT. NCDB analyses are also limited by certain covariates coded by specific billing and diagnosis codes. Due to the above limitations, NCDB analyses should be used for hypothesis generation as positive findings often suggest association instead of causation. Ultimately, a randomized controlled trial comparing radical cystectomy vs definitive chemoRT is needed.

A recently published NCDB analyses defined definitive chemoRT as radiation doses ≥40 Gy (median radiation dose 45 Gy) and performed a propensity score matched pair analyses using clinical T‐stage (instead of overall clinical stage) grouping into cT2 vs cT3‐cT4.^20^ Ritch et al. showed that overall survival was better after cystectomy than definitive chemoRT, which is opposite of what this study identified. Possible explanations for this difference include the low radiation dose used to specify curative radiotherapy (median dose 45 Gy is not a definitive radiotherapy dose as NCCN recommends ≥55 Gy), matching using clinical T stage instead of clinical overall stage, and differences in the tolerance score chosen for matching (this report used propensity score of 0 resulting in exact matching).^6^ In this report, the mean and median radiotherapy dose in chemoRT patients was 64.8 Gy, and radiation dose was significantly correlated with improved overall survival (Figure 5).

We also investigated the effect of chemotherapy on overall survival. Our finding that radical cystectomy patients treated with neoadjuvant chemotherapy had better overall survival compared to ones who received adjuvant chemotherapy was consistent with a previous publication.^27^ We also found a radiation dose‐response as ≥60 Gy resulted in better overall survival than 55 Gy to <60 Gy. To our knowledge, this is the first report that identified a radiation dose‐response for bladder cancer using the NCDB. The importance of radiation dose might explain why Ritch et al.^20^ showed inferior overall survival with definitive chemoRT compared to cystectomy when ≥40 Gy was used to specify definitive chemoRT.

To date, there has not been a prospective randomized trial comparing radical cystectomy and bladder preservation therapy with definitive chemoRT. However, results from prospective randomized radiotherapy trials have shown overall survival that is comparable to radical cystectomy. A pooled analysis of long‐term outcomes from several prospective randomized and nonrandomized RTOG trials showed a 5‐year overall survival of 57% and 5‐year disease‐specific survival of 71%.^12^ This overall survival rate compared favorably with the 5‐year overall survival rate of 50% among cystectomy patients treated with platinum combination neoadjuvant chemotherapy in a meta‐analysis.^15^ Similarly, Grossman et al^9^ reported a 5‐year overall survival rate of 57% for patients treated with neoadjuvant MVAC (methotrexate, vinblastine, doxorubicin, and cisplatin) followed by radical cystectomy. Single‐institution prospective definitive radiotherapy trials have also shown comparable 5‐year overall survival of 57%.^28^ For a subset of patients who responded favorably to induction chemoRT with a complete or near‐complete response, the 5‐year overall survival could be as high as 61% (near‐complete response to induction chemoRT) to 72% (complete response to induction chemoRT).^13^

## 5 CONCLUSION

In the National Cancer Database, patients treated with cystectomy/chemo were significantly younger and had better performance status compared to those who received definitive chemoRT. When accounting for these factors, in addition to the nearly 30% probability of upstaging following cystectomy, any survival advantage associated with radical surgery relative to organ preservation with chemoRT disappeared. In the absence of a randomized trial, this analysis contributes further support to the use of organ preservation with combination chemoRT as a viable option in the management of muscle‐invasive bladder cancer. Optimal patient selection for bladder preservation therapy needs further investigation.

## CONFLICT OF INTEREST

None of the authors have direct or indirect commercial financial incentives associated with the publication of this article.
