# Supplementary figures and images for: National Cancer Database Comparison of Radical Cystectomy vs Chemoradiotherapy for Muscle‐Invasive Bladder Cancer: Implications of Using Clinical vs Pathologic Staging
Source: Cancer Med. 2018 Oct 10;7(11):5370–81. doi: 10.1002/cam4.1684 (PMC6247074; doi:10.1002/cam4.1684)

Supplemental Figure 1A

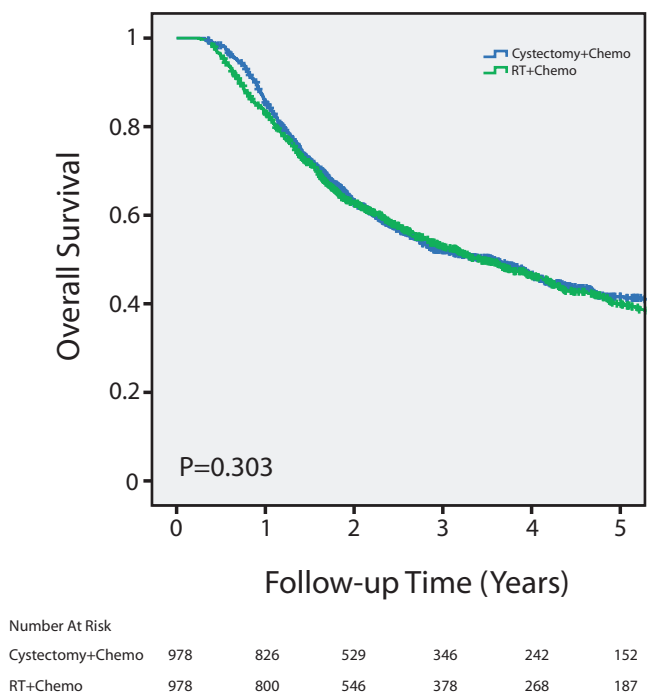

Supplemental Figure 1B

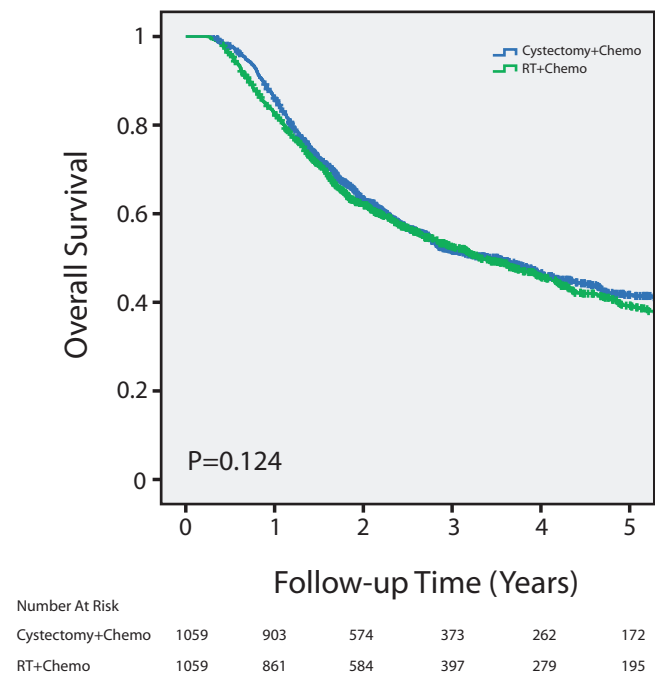

Supplemental Figure 1C

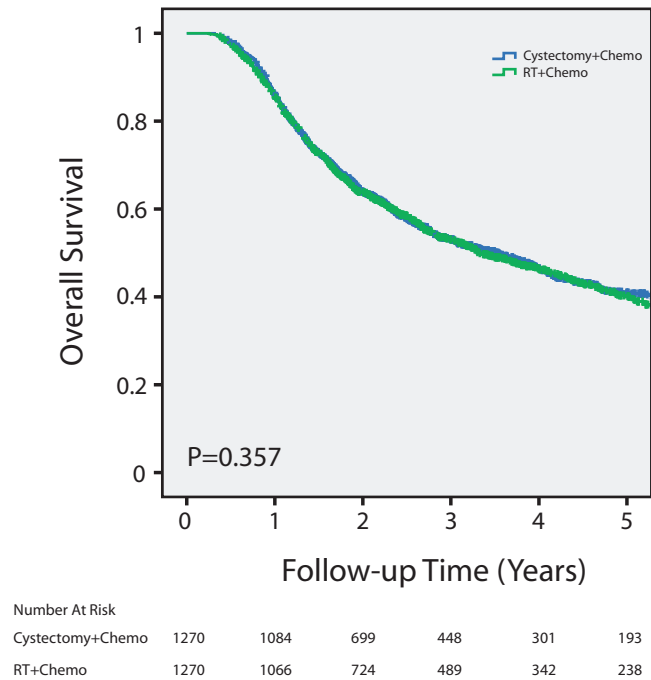

Supplement: Supplementary file 1 [file CAM4-7-5370-s001.pdf]
