# Supplementary material for: National Cancer Database Comparison of Radical Cystectomy vs Chemoradiotherapy for Muscle‐Invasive Bladder Cancer: Implications of Using Clinical vs Pathologic Staging
Source: Cancer Med. 2018 Oct 10;7(11):5370–81. doi: 10.1002/cam4.1684 (PMC6247074; doi:10.1002/cam4.1684)
Supplement: Supplementary file 2 [file CAM4-7-5370-s002.docx]

**Supplementary Table 1A** Patient Characteristics After Matched Pair Analyses Using Clinical Stage

|  | Cystectomy and Chemo | RT and Chemo | P Value |
| --- | --- | --- | --- |
| N | 875 | 875 |  |
| Age |  |  | 1.000 |
| Mean | 71.67 | 71.67 |  |
| Race |  |  | 1.000 |
| White | 855 (97.7%) | 855 (97.7%) |  |
| Black | 16 (1.8%) | 16 (1.8%) |  |
| Other/Unknown | 4 (0.5%) | 4 (0.5%) |  |
| Sex |  |  | 1.000 |
| Male | 680 (77.7%) | 680 (77.7%) |  |
| Female | 195 (22.3%) | 195 (22.3%) |  |
| CDCS |  |  | 1.000 |
| 0 | 628 (71.8%) | 628 (71.8%) |  |
| 1 | 204 (23.3%) | 204 (23.3%) |  |
| 2 or more | 43 (4.9%) | 43 (4.9%) |  |
| Clinical Stage |  |  | 1.000 |
| II | 707 (80.8%) | 707 (80.8%) |  |
| III | 112 (12.8%) | 112 (12.8%) |  |
| IV | 56 (6.4%) | 56 (6.4%) |  |
| Facility Type |  |  | 1.000 |
| Academic/Research Program | 228 (26.1%) | 228 (26.1%) |  |
| Non-Academic/Research Program | 647 (73.9%) | 647 (73.9%) |  |
| Insurance |  |  | 1.000 |
| Private | 174 (19.9%) | 174 (19.9%) |  |
| Public | 689 (78.7%) | 689 (78.7%) |  |
| Uninsured | 12 (1.4%) | 12 (1.4%) |  |

**Supplementary Table 1B** Patient Characteristics After Matched Pair Analyses Using Analytic Stage

|  | Cystectomy and Chemo | RT and Chemo | P Value |
| --- | --- | --- | --- |
| N | 734 | 734 |  |
| Age |  |  | 1.000 |
| Mean | 71.04 | 71.04 |  |
| Race |  |  | 1.000 |
| White | 721 (98.2%) | 721 (98.2%) |  |
| Black | 10 (1.4%) | 10 (1.4%) |  |
| Other/Unknown | 3 (0.4%) | 3 (0.4%) |  |
| Sex |  |  | 1.000 |
| Male | 586 (79.8%) | 586 (79.8%) |  |
| Female | 148 (20.2%) | 148 (20.2%) |  |
| CDCS |  |  | 1.000 |
| 0 | 549 (74.8%) | 549 (74.8%) |  |
| 1 | 158 (21.5%) | 158 (21.5%) |  |
| 2 or more | 27 (3.7%) | 27 (3.7%) |  |
| Analytic Stage |  |  | 1.000 |
| II | 514 (70.0%) | 514 (70.0%) |  |
| III | 132 (18.0%) | 132 (18.0%) |  |
| IV | 88 (12.0%) | 88 (12.0%) |  |
| Facility Type |  |  | 1.000 |
| Academic/Research Program | 534 (72.8%) | 534 (72.8%) |  |
| Non-Academic/Research Program | 200 (27.2%) | 200 (27.2%) |  |
| Insurance |  |  | 1.000 |
| Private | 156 (21.3%) | 156 (21.3%) |  |
| Public | 568 (77.4%) | 568 (77.4%) |  |
| Uninsured | 10 (1.4%) | 10 (1.4%) |  |
