# Supplementary material for: National Cancer Database Comparison of Radical Cystectomy vs Chemoradiotherapy for Muscle‐Invasive Bladder Cancer: Implications of Using Clinical vs Pathologic Staging
Source: Cancer Med. 2018 Oct 10;7(11):5370–81. doi: 10.1002/cam4.1684 (PMC6247074; doi:10.1002/cam4.1684)
Supplement: Supplementary file 3 [file CAM4-7-5370-s003.docx]

**Supplementary Table 2A.** Univariate and Multivariate Analyses of Patient Variables Correlated with Overall Survival after Matched Pair Analyses using Clinical Stage

|  | Univariate |  | Multivariate |  |
| --- | --- | --- | --- | --- |
|  | P Value | Hazard Ratio (95% CI) | P Value | Hazard Ratio (95% CI) |
| Age | **<0.001** | 1.015 (1.008-1.023) | **0.001** | 1.016 (1.006-1.026) |
| Sex (reference: Male)  Female vs. Male | 0.463 | 1.057 (0.910-1.227) | 0.716 | 1.029 (0.881-1.203) |
| Race (reference: White)  Black vs. White  Other vs. White  Unknown vs. White | 0.063  0.251  0.678 | 1.539 (0.977-2.424)  1.779 (0.666-4.751)  0.666 (0.093-4.696) | **0.002**  0.182  **0.036** | 2.055 (1.293-3.257)  1.960 (0.730-5.266)  8.236 (1.147-59.119) |
| Charlson Deyo Comorbidity Score  (reference: 0)  1 vs. 0  ≥2 vs. 0 | 0.085  **0.011** | 1.140 (0.982-1.323)  1.417 (1.083-1.853) | **0.021**  **0.002** | 1.195 (1.027-1.389)  1.548 (1.178-2.035) |
| Clinical Stage (reference: II)  III vs. II  IV vs. II | **<0.001**  **<0.001** | 1.513 (1.268-1.805)  1.982 (1.581-2.485) | **<0.001**  **<0.001** | 1.544 (1.287-1.852)  2.141 (1.555-2.517) |
| Treatment Modality  (reference: Cystectomy/Chemo)  ChemoRT vs. Cystectomy/Chemo | 0.501 | 1.044 (0.921 -1.185) | 0.422 | 1.054 (0.927-1.199) |
| Facility Type (reference: Non-Academic)  Academic vs. Non-Academic | 0.370 | 0.935 (0.808-1.083) | 0.443 | 0.944 (0.815-1.094) |
| Insurance  (reference: Not Insured/Unknown)  Private vs. Not Insured/Unknown  Government vs. Not Insured/Unknown | 0.479  0.913 | 0.816 (0.464-1.434)  1.031 (0.596 -1.784) | 0.105  0.151 | 0.623 (0.352-1.104)  0.656 (0.369-1.166) |
| Income (reference: <$30,000)  $30,000-$34,999 vs. <$30,000  $35,000-$45,999 vs. <$30,000  ≥$46,000 vs. <$30,000 | **0.046**  **0.019**  0.471 | 1.302 (1.005-1.686)  1.342 (1.049-1.715)  1.093 (0.859-1.391) | 0.073  0.072  0.750 | 1.269 (0.978-1.647)  1.255 (0.979-1.609)  1.040 (0.816-1.327) |

**Supplementary Table 2B.** Univariate and Multivariate Analyses of Patient Variables Correlated with Overall Survival after Matched Pair Analyses using Analytic Stage

|  | Univariate |  | Multivariate |  |
| --- | --- | --- | --- | --- |
|  | P Value | Hazard Ratio (95%CI) | P Value | Hazard Ratio (95%CI) |
| Age | **<0.001** | 1.027 (1.017-1.036) | **<0.001** | 1.025 (1.014-1.037) |
| Sex (reference: Male)  Female vs. Male | 0.273 | 1.103 (0.925-1.316) | 0.291 | 1.104 (0.919-1.325) |
| Race (reference: White)  Black vs. White  Other vs. White  Unknown vs. White | 0.063  0.527  0.752 | 1.719 (0.971-3.042)  0.531 (0.075-3.773)  0.729 (0.102-5.189) | **0.006**  0.328  **0.020** | 2.274 (1.270-4.074)  0.374 (0.052-2.685)  10.419 (1.445-75.104) |
| Charlson Deyo Comorbidity Score  (reference: 0)  1 vs. 0  ≥2 vs. 0 | 0.079  **0.003** | 1.169 (0.982-1.392)  1.660 (1.189-2.317) | **0.007**  **0.001** | 1.284 (1.072-1.538)  1.836 (1.300-2.593) |
| Analytic Stage (reference: II)  III vs. II  IV vs. II | **<0.001**  **<0.001** | 1.891 (1.581-2.262)  2.769 (2.283-3.358) | **<0.001**  **<0.001** | 1.790 (1.485-2.158)  2.714 (2.221-3.318) |
| Treatment Modality  (reference: Cystectomy/Chemo)  RT Chemo vs. Cystectomy Chemo | **<0.001** | 1.4583 (1.256-1.682) | **<0.001** | 1.453 (1.252-1.687) |
| Facility Type (reference: Non-Academic)  Academic vs. Non-Academic | 0.910 | 0.991 (0.842-1.166) | 0.211 | 1.112 (0.941-1.315) |
| Insurance  (reference: Not Insured/Unknown)  Private vs. Not Insured/Unknown  Government vs. Not Insured/Unknown | 0.437  0.824 | 0.765 (0.389-1.503)  1.078 (0.558-2.082) | 0.082  0.080 | 0.545 (0.275-1.080)  0.537 (0.268-1.078) |
| Income (reference: <$30,000)  $30,000-$34,999 vs. <$30,000  $35,000-$45,999 vs. <$30,000  ≥$46,000 vs. <$30,000 | 0.347  0.312  0.608 | 1.149 (0.861-1.533)  1.148 (0.878-1.502)  1.071 (0.824-1.392) | 0.122  0.108  0.567 | 1.259 (0.941-1.685)  1.249 (0.953-1.636)  1.080 (0.829-1.407) |
