# Supplementary material for: National Cancer Database Comparison of Radical Cystectomy vs Chemoradiotherapy for Muscle‐Invasive Bladder Cancer: Implications of Using Clinical vs Pathologic Staging
Source: Cancer Med. 2018 Oct 10;7(11):5370–81. doi: 10.1002/cam4.1684 (PMC6247074; doi:10.1002/cam4.1684)
Supplement: Supplementary file 4 [file CAM4-7-5370-s004.docx]

**Supplementary Table 3A** Matched Pair Analyses Using Less Stringent Matching Criteria

|  | Cystectomy and Chemo | RT and Chemo | P Value |
| --- | --- | --- | --- |
| N | 978 | 978 |  |
| Age |  |  | 1.000 |
| Mean | 71.54 | 71.54 |  |
| Race |  |  | **<0.001** |
| White | 909 (92.9%) | 870 (89.0%) |  |
| Black | 32 (3.3%) | 80 (8.2%) |  |
| Other/Unknown | 37 (3.8%) | 28 (2.8%) |  |
| Sex |  |  | 1.000 |
| Male | 748 (76.5%) | 748 (76.5%) |  |
| Female | 230 (23.5%) | 230 (23.5%) |  |
| CDCS |  |  | 1.000 |
| 0 | 697 (71.3%) | 697 (71.3%) |  |
| 1 | 227 (23.2%) | 227 (23.2%) |  |
| 2 or more | 54 (5.5%) | 54 (5.5%) |  |
| Clinical Stage |  |  | 1.000 |
| II | 786 (80.4%) | 786 (80.4%) |  |
| III | 123 (12.6%) | 123 (12.6%) |  |
| IV | 69 (7.1%) | 69 (7.1%) |  |
| Facility Type |  |  | 1.000 |
| Academic | 705 (72.1%) | 705 (72.1%) |  |
| Non-Academic | 273 (27.9%) | 273 (27.9%) |  |
| Insurance |  |  | 1.000 |
| Private | 201 (20.6%) | 201 (20.6%) |  |
| Public | 760 (77.7%) | 760 (77.7%) |  |
| Uninsured/Unknown | 17 (1.7%) | 17 (1.7%) |  |

**Supplementary Table 3B** Matched Pair Analyses Using Less Stringent Matching Criteria

|  | Cystectomy and Chemo | RT and Chemo | P Value |
| --- | --- | --- | --- |
| N | 1059 | 1059 |  |
| Age |  |  | 1.000 |
| Mean | 71.73 | 71.73 |  |
| Race |  |  | **0.004** |
| White | 971 (91.7%) | 941 (88.9%) |  |
| Black | 45 (4.2%) | 84 (7.9%) |  |
| Other/Unknown | 43 (4.1%) | 34 (3.2%) |  |
| Sex |  |  | **<0.001** |
| Male | 698 (65.9%) | 819 (77.3%) |  |
| Female | 361 (34.1%) | 240 (22.7%) |  |
| CDCS |  |  | 1.000 |
| 0 | 743 (70.2%) | 743 (70.2%) |  |
| 1 | 252 (23.8%) | 252 (23.8%) |  |
| 2 or more | 64 (6.0%) | 64 (6.0%) |  |
| Clinical Stage |  |  | 1.000 |
| II | 833 (78.7%) | 833 (78.7%) |  |
| III | 142 (13.4%) | 142 (13.4%) |  |
| IV | 84 (7.9%) | 84 (7.9%) |  |
| Facility Type |  |  | 1.000 |
| Academic | 764 (72.1%) | 764 (72.1%) |  |
| Non-Academic | 295 (27.9%) | 295 (27.9%) |  |
| Insurance |  |  | 1.000 |
| Private | 212 (20.0%) | 212 (20.0%) |  |
| Public | 824 (77.8%) | 824 (77.8%) |  |
| Uninsured/Unknown | 23 (2.2%) | 23 (2.2%) |  |

**Supplementary Table 3C** Matched Pair Analyses Using Less Stringent Matching Criteria

|  | Cystectomy and Chemo | RT and Chemo | P Value |
| --- | --- | --- | --- |
| N | 1270 | 1270 |  |
| Age |  |  | 1.000 |
| Mean | 70.43 | 70.43 |  |
| Race |  |  | 1.000 |
| White | 1241 (97.7%) | 1241 (97.7%) |  |
| Black | 23 (1.8%) | 23 (1.8%) |  |
| Other/Unknown | 6 (0.5%) | 6 (0.5%) |  |
| Sex |  |  | 1.000 |
| Male | 1004 (79.1%) | 1004 (79.1%) |  |
| Female | 266 (20.9%) | 266 (20.9%) |  |
| CDCS |  |  | 1.000 |
| 0 | 914 (72.0%) | 914 (72.0%) |  |
| 1 | 298 (23.5%) | 298 (23.5%) |  |
| 2 or more | 58 (4.6%) | 58 (4.6%) |  |
| Clinical Stage |  |  | 1.000 |
| II | 995 (78.3%) | 995 (78.3%) |  |
| III | 162 (12.8%) | 162 (12.8%) |  |
| IV | 113 (8.9%) | 113 (8.9%) |  |
| Facility Type |  |  | 1.000 |
| Academic/Research Program | 909 (71.6%) | 909 (71.6%) |  |
| Non-Academic/Research Program | 361 (28.4%) | 361 (28.4%) |  |
| Insurance |  |  | 1.000 |
| Private | 279 (22.0%) | 279 (22.0%) |  |
| Public | 971 (76.5%) | 971 (76.5%) |  |
| Uninsured | 20 (1.6%) | 20 (1.6%) |  |
