# Supplementary material for: National Cancer Database Comparison of Radical Cystectomy vs Chemoradiotherapy for Muscle‐Invasive Bladder Cancer: Implications of Using Clinical vs Pathologic Staging
Source: Cancer Med. 2018 Oct 10;7(11):5370–81. doi: 10.1002/cam4.1684 (PMC6247074; doi:10.1002/cam4.1684)
Supplement: Supplementary file 5 [file CAM4-7-5370-s005.docx]

**Supplemental Table 4A** Definitive ChemoRT Patient Characteristics According to Radiation Dose Before Matched Pair Analyses

|  | 55Gy to 59.9Gy | ≥60Gy | P Value |
| --- | --- | --- | --- |
| N | 203 | 1455 |  |
| Age |  |  | **0.002** |
| Mean | 77.1 | 74.9 |  |
| Race |  |  | 0.974 |
| White | 181 (89.1%) | 1309 (90.0%) |  |
| Black | 16 (7.9%) | 101 (6.9%) |  |
| Other/Unknown | 6 (3.0%) | 45 (3.1%) |  |
| Sex |  |  | **0.002** |
| Male | 135 (66.5%) | 1116 (76.7%) |  |
| Female | 68 (33.5%) | 339 (23.3%) |  |
| CDCS |  |  | 0.260 |
| 0 | 139 (68.5%) | 958 (65.8%) |  |
| 1 | 40 (19.7%) | 356 (24.5%) |  |
| 2 or more | 24 (11.8%) | 141 (9.7%) |  |
| Clinical Stage |  |  | 0.157 |
| II | 145 (71.4%) | 1127 (77.5%) |  |
| III | 37 (18.2%) | 215 (14.8%) |  |
| IV | 21 (10.3%) | 113 (7.8%) |  |
| Facility Type |  |  | 0.200 |
| Academic/Research Program | 58 (28.6%) | 351 (24.1%) |  |
| Non-Academic/Research Program | 145 (71.4%) | 1104 (75.9%) |  |
| Insurance |  |  | 0.563 |
| Private | 33 (16.3%) | 264 (18.1%) |  |
| Public | 166 (81.8%) | 1148 (78.9%) |  |
| Uninsured | 4 (2.0%) | 43 (3.0%) |  |
| Income |  |  | 0.366 |
| <$30,000 | 21 (10.3%) | 168 (11.5%) |  |
| $30,000 -- $34,999 | 31 (15.3%) | 283 (19.5%) |  |
| $35,000 -- $45,999 | 62 (30.5%) | 405 (27.8%) |  |
| ≥$46,000 | 85 (41.9%) | 544 (37.4%) |  |
| Unknown | 4 (2.0%) | 55 (3.8%) |  |

**Supplemental Table 4B** Definitive ChemoRT Patient Characteristics According to Radiation Dose After Matched Pair Analyses

|  | 55Gy – 59.9Gy | ≥60Gy | P Value |
| --- | --- | --- | --- |
| N | 202 | 202 |  |
| Age |  |  | 1.000 |
| Mean | 77.3 | 77.3 |  |
| Race |  |  | 0.938 |
| White | 180 (89.1%) | 178 (88.1%) |  |
| Black | 16 (7.9%) | 19 (9.4%) |  |
| Other/Unknown | 6 (3.0%) | 5 (2.5%) |  |
| Sex |  |  | 1.000 |
| Male | 135 (66.8%) | 135 (66.8%) |  |
| Female | 67 (33.2%) | 67 (33.2%) |  |
| CDCS |  |  | 0.885 |
| 0 | 138 (68.3%) | 135 (66.8%) |  |
| 1 | 40 (19.8%) | 44 (21.8%) |  |
| 2 or more | 24 (11.9%) | 23 (11.4%) |  |
| Clinical Stage |  |  | 0.924 |
| II | 145 (71.8%) | 145 (71.8%) |  |
| III | 37 (18.3%) | 39 (19.3%) |  |
| IV | 20 (9.9%) | 18 (8.9%) |  |
| Facility Type |  |  | 0.065 |
| Academic/Research Program | 58 (28.7%) | 42 (20.8%) |  |
| Non-Academic/Research Program | 144 (71.3%) | 160 (79.2%) |  |
| Insurance |  |  | 0.922 |
| Private | 32 (15.8%) | 35 (17.3%) |  |
| Public | 166 (82.2%) | 163 (80.7%) |  |
| Uninsured | 4 (2.0%) | 4 (2.0%) |  |
| Income |  |  | 0.328 |
| <$30,000 | 26 (12.8%) | 26 (12.8%) |  |
| $30,000 -- $34,999 | 43 (21.3%) | 38 (18.8%) |  |
| $35,000 -- $45,999 | 48 (23.8%) | 63 (31.2%) |  |
| ≥$46,000 | 81 (40.1%) | 68 (33.7%) |  |
| Unknown | 4 (2.0%) | 7 (3.5%) |  |
