# Supplementary material for: National Cancer Database Comparison of Radical Cystectomy vs Chemoradiotherapy for Muscle‐Invasive Bladder Cancer: Implications of Using Clinical vs Pathologic Staging
Source: Cancer Med. 2018 Oct 10;7(11):5370–81. doi: 10.1002/cam4.1684 (PMC6247074; doi:10.1002/cam4.1684)
Supplement: Supplementary file 6 [file CAM4-7-5370-s006.docx]

**Supplemental Figure 1.** Survival after matched pair analyses using progressively less stringent matching criteria. Despite using progressively less stringent matching criteria, all matched pair analyses continued to show similar overall survival between cystectomy/chemo and chemoRT. Chemo, chemotherapy. RT, radiotherapy.
